# Identification of sonic hedgehog-regulated genes and biological processes in the cranial neural crest mesenchyme by comparative transcriptomics

**DOI:** 10.1186/s12864-018-4885-5

**Published:** 2018-06-27

**Authors:** Joshua L. Everson, Dustin M. Fink, Hannah M. Chung, Miranda R. Sun, Robert J. Lipinski

**Affiliations:** 1 Department of Comparative Biosciences, School of Veterinary Medicine, University of Wisconsin-Madison, 2015 Linden Dr., Madison, WI 53706 USA; 2 Molecular and Environmental Toxicology Center, University of Wisconsin-Madison, Madison, WI 53706 USA

**Keywords:** Sonic hedgehog, Hedgehog target gene, Cranial neural crest cell, Frontonasal prominence, Medial nasal process, Orofacial development, Cleft lip, Comparative transcriptomics

## Abstract

**Background:**

The evolutionarily conserved Sonic Hedgehog (Shh) signaling pathway is essential for embryogenesis and orofacial development. SHH ligand secreted from the surface ectoderm activates pathway activity in the underlying cranial neural crest cell (cNCC)-derived mesenchyme of the developing upper lip and palate. Disruption of Shh signaling causes orofacial clefts, but the biological action of Shh signaling and the full set of Shh target genes that mediate normal and abnormal orofacial morphogenesis have not been described.

**Results:**

Using comparative transcriptional profiling, we have defined the Shh-regulated genes of the cNCC-derived mesenchyme. Enrichment analysis demonstrated that in cultured cNCCs, Shh-regulated genes are involved in smooth muscle and chondrocyte differentiation, as well as regulation of the Forkhead family of transcription factors, G1/S cell cycle transition, and angiogenesis. Next, this gene set from Shh-activated cNCCs in vitro was compared to the set of genes dysregulated in the facial primordia in vivo during the initial pathogenesis of Shh pathway inhibitor-induced orofacial clefting. Functional gene annotation enrichment analysis of the 112 Shh-regulated genes with concordant expression changes linked Shh signaling to interdependent and unique biological processes including mesenchyme development, cell adhesion, cell proliferation, cell migration, angiogenesis, perivascular cell markers, and orofacial clefting.

**Conclusions:**

We defined the Shh-regulated transcriptome of the cNCC-derived mesenchyme by comparing the expression signatures of Shh-activated cNCCs in vitro to primordial midfacial tissues exposed to the Shh pathway inhibitor in vivo. In addition to improving our understanding of cNCC biology by determining the identity and possible roles of cNCC-specific Shh target genes**,** this study presents novel candidate genes whose examination in the context of human orofacial clefting etiology is warranted.

**Electronic supplementary material:**

The online version of this article (10.1186/s12864-018-4885-5) contains supplementary material, which is available to authorized users.

## Background

Orofacial clefts (OFCs) are the most common human craniofacial birth defects, occurring in 1–2 per 1000 live births [1]. Although the causes of OFCs are unknown for most cases and likely involve multifactorial gene-environment interactions [2–6], evidence from humans indicates a strong genetic component. For example, the prevalence of OFCs differs between races and ethnicities [1]. In addition, while the overall frequency of the most prevalent form of OFCs in humans, cleft lip with or without cleft palate, is approximately 0.1% in the general population, concurrence rates in monozygotic twins, dizygotic twins, and siblings are 35–50, 5%, and 3–5%, respectively [4, 7].

**Fig. 1:**
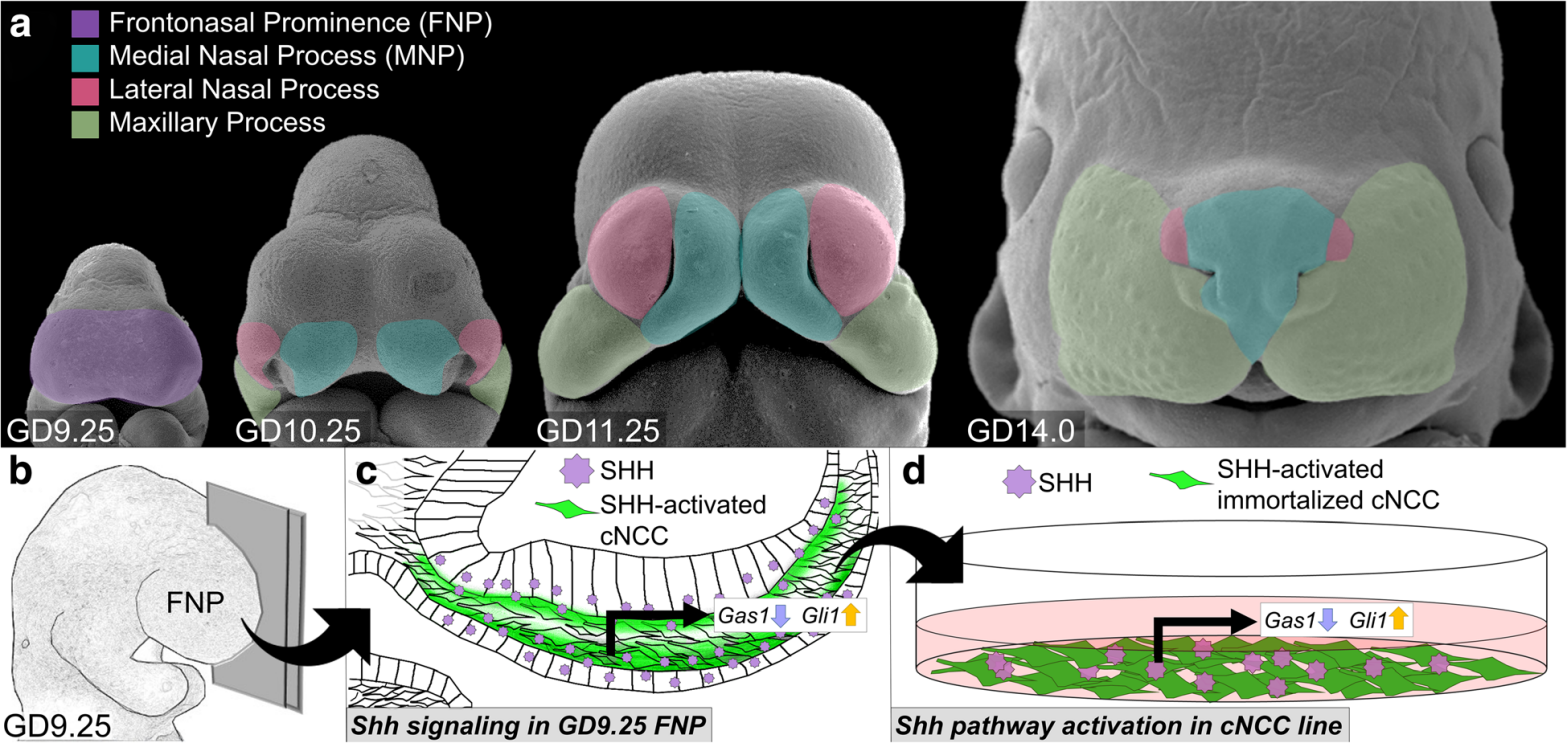
Schematic of Shh pathway activation in cNCCs in vivo and in vitro*.* **a** Pseudo-colored scanning electron micrographs of GD9.25, 10.25, 11.25, and 14.0 mouse embryos show the dynamic morphological changes of orofacial development. Embryos not in exact scale. **b** Schematic of GD9.25 embryo being cut mid-sagittally to reveal the cross-section in (**c**). **c** Schematic showing normal Shh pathway activity in vivo in GD9.25 FNP tissue*.* SHH ligand secreted from the surface and neural ectoderm activates post-migratory cNCC-derived mesenchymal cells in facial primordia (green shading), leading to regulation of Shh target genes, like *Gli1* (positively regulated by Shh signaling) and *Gas1* (negatively regulated by Shh signaling). **d** Schematic of in vitro cNCC Shh pathway activation by addition of SHH ligand, which alters expression of Shh-regulated genes in the SHH-responding cNCCs

Orofacial development is highly conserved in mammals, requiring the coordinated growth and fusion of the embryonic facial primordia [8]. At gestational day (GD)9.25 in the mouse, the frontonasal prominence (FNP) is evident at the anterior of the embryo and will give rise to the paired medial nasal processes (MNPs) and lateral nasal processes by GD10.25 (Fig. 1a). The MNPs continue to grow distolaterally until apposition and fusion with the maxillary processes at GD11.25, closing the upper lip, as visible at GD14 (Fig. 1a). Failure of these growth and fusion processes results in OFCs [8].

The embryonic facial primordia that will form the upper lip and midface, the FNP and MNPs, are comprised of densely packed, multipotent cranial neural crest cell (cNCC)-derived mesenchyme overlaid by surface ectoderm. During orofacial development, the surface ectoderm provides important morphogenic stimuli to the underlying cNCC-derived mesenchyme of the FNP and MNPs, including Sonic hedgehog ligand (SHH) [9]. Previous work by our group has shown that perturbation of Shh signaling during early craniofacial development disrupts proliferation of the cNCC-derived mesenchyme of the MNPs, resulting in OFC phenotypes that recapitulate outcomes commonly observed in children with OFCs [10].

The Shh pathway is a conserved morphogenic signal transduction cascade that is critical for the epithelial-mesenchymal interactions that drive orofacial development. SHH is secreted from the surface ectoderm and binds cell surface receptors on the underlying cNCC-derived mesenchymal cells, resulting in the de-repression of the transmembrane protein Smoothened and modulation of Shh target gene expression via the three conserved Gli transcription factors (Fig. 1b, c) [11]. The Shh/Smoothened signaling pathway is necessary for orofacial development, but the complete complement of pathway target genes and their biological functions are not known.

Here, comparative transcriptomics was used to identify Shh target genes in the cNCC-derived orofacial mesenchyme. The transcriptional response of immortalized cNCCs stimulated with SHH ligand was compared to the response of GD9.25 FNP tissue acutely exposed to the Shh pathway inhibitor cyclopamine in vivo. Integration of these complementary systems allowed for the identification of cNCC-specific Shh target genes, which are also dysregulated during the initial pathogenesis of orofacial clefting.

## Results

### Definition of the Shh-regulated cNCC transcriptome

**Fig. 2:**
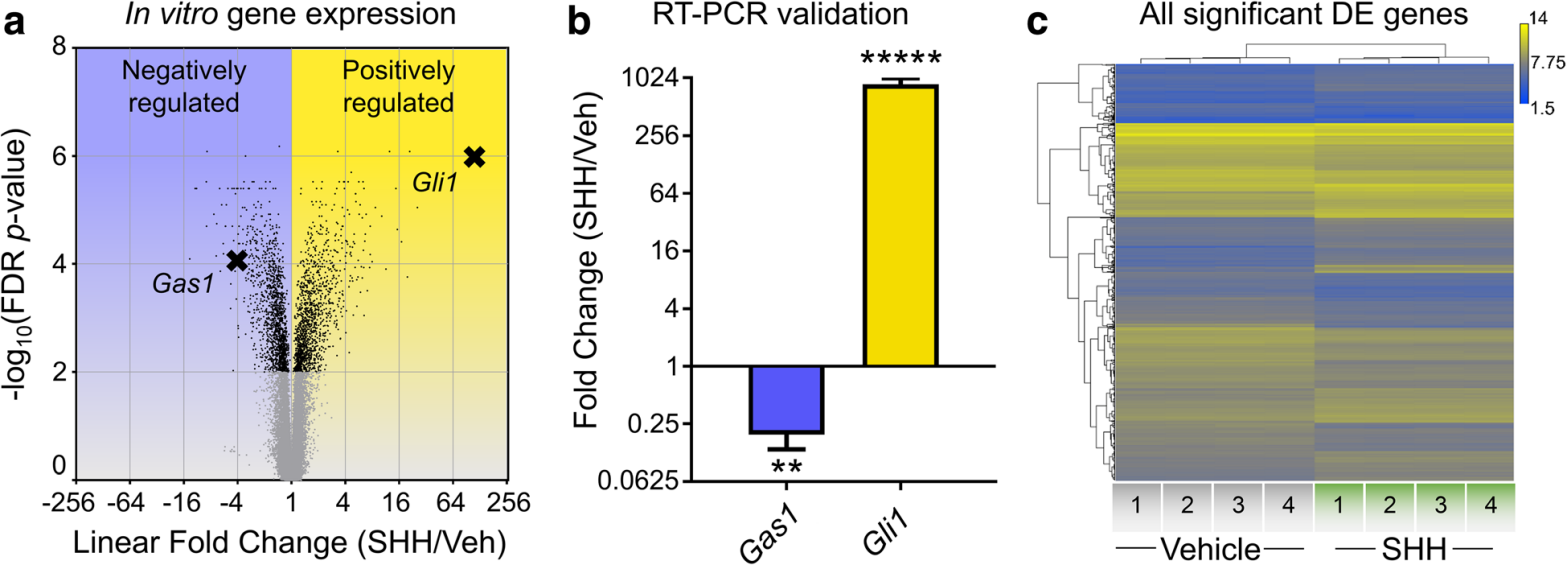
Identification of Shh-regulated genes in cNCCs in vitro. **a** Volcano plot showing the linear fold change (SHH/Veh) (x-coordinate) and –log_10_ FDR *p*-value (y-coordinate) of each differentially expressed gene. Black dots represent significant differentially expressed genes (FDR *p*-value < 0.01), and grey dots mark genes with non-significant changes. The bona fide Shh target genes *Gas1* (negatively regulated) and *Gli1* (positively regulated) are labeled and marked with X’s. **b** RT-PCR validated the downregulation of *Gas1* and upregulation of *Gli1* in SHH-activated cNCCs (t-test, ** *p*-value < 0.015, ***** *p*-value < 0.00001). **c** Hierarchical clustering heat map of 2749 significant differentially expressed genes (FDR *p*-value < 0.01) between vehicle- and SHH-treated cNCCs shows the robust differential expression and high level of consistency within treatments for the in vitro model. Heat map represents log_10_ signal intensity for each gene in each condition, which are then clustered by biological response. Yellow represents genes with higher expression while blue represents genes with lower expression. *N* = 4 biological replicates (1–4)

**Fig. 3:**
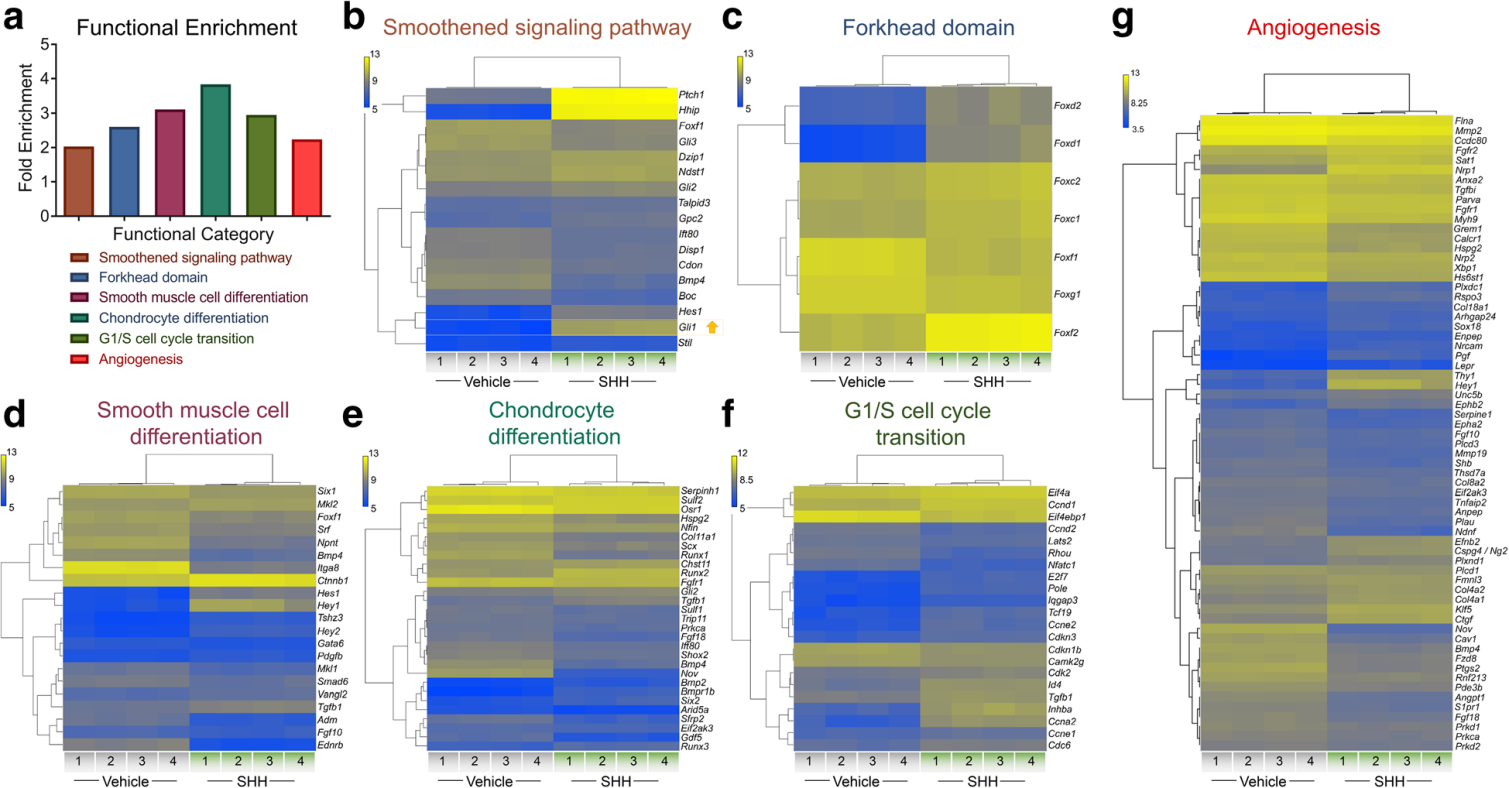
Enrichment analysis of Shh-regulated genes in cNCCs in vitro. **a** Functional enrichment analysis of 2749 genes that are significantly dysregulated in cNCCs stimulated by SHH in vitro*.* Each selected functional category is significantly enriched within the differentially expressed gene set. **b**-**g** Hierarchically clustered heat maps for significantly enriched biological categories showing the specific significantly dysregulated genes (FDR *p*-value < 0.01) in each biological category. *Gli1* is highlighted in (**b**). Heat maps represent log_10_ signal intensity for each gene in each condition, which are then clustered by biological response. Yellow represents genes with higher expression while blue represents genes with lower expression. *N* = 4 biological replicates (1–4)

To define the Shh-regulated transcriptome of the cNCC-derived mesenchyme, we utilized an immortalized cNCC line (O9–1) that maintains the transcriptional profile and multipotency of the post-migrational cNCC-derived mesenchyme [12]. Shh ligand (SHH) or vehicle-only was added to cNCCs in vitro*,* and 48 h later gene expression was assessed by microarray (Fig. 1d). SHH stimulation of cNCCs resulted in the differential expression of 2749 genes, with 1305 genes upregulated and 1444 genes downregulated (Fig. 2a and Additional file 1). The differential expression of the bona fide Shh-target genes *Gli1* (positively regulated) and *Gas1* (negatively regulated) was verified by RT-PCR (Fig. 2b). Hierarchical clustering analysis demonstrated up- and downregulation of 2749 significant differentially expressed genes (Fig. 2c). To better understand the biological roles of the Shh-regulated genes of the cNCC transcriptome, functional annotation enrichment analysis was conducted on the 2749 significant differentially expressed gene set (Fig. 3a and Additional file 2). As expected, Shh pathway genes (Smoothened signaling pathway) were significantly enriched within the differentially expressed gene set (fold enrichment = 2.029, *p*-value = 5.61 × 10^− 3^) (Fig. 3b). Significant enrichment was also observed for several biological processes with known or proposed roles in cNCC biology and orofacial development, including smooth muscle differentiation (fold enrichment: 3.113, *p*-value = 1.84 × 10^− 2^), chondrocyte differentiation (fold enrichment = 3.828, *p*-value = 2.95 × 10^− 8^), G1/S cell cycle transition (fold enrichment = 2.949, *p*-value = 2.42 × 10^− 2^), Forkhead domain (fold enrichment = 2.602, *p*-value = 1.75 × 10^− 2^), and angiogenesis (fold enrichment = 2.24, *p*-value = 2.11 × 10^− 10^) (Fig. 3c-g).

### Comparative analysis of Shh-regulated genes in vitro and in vivo

**Fig. 4:**
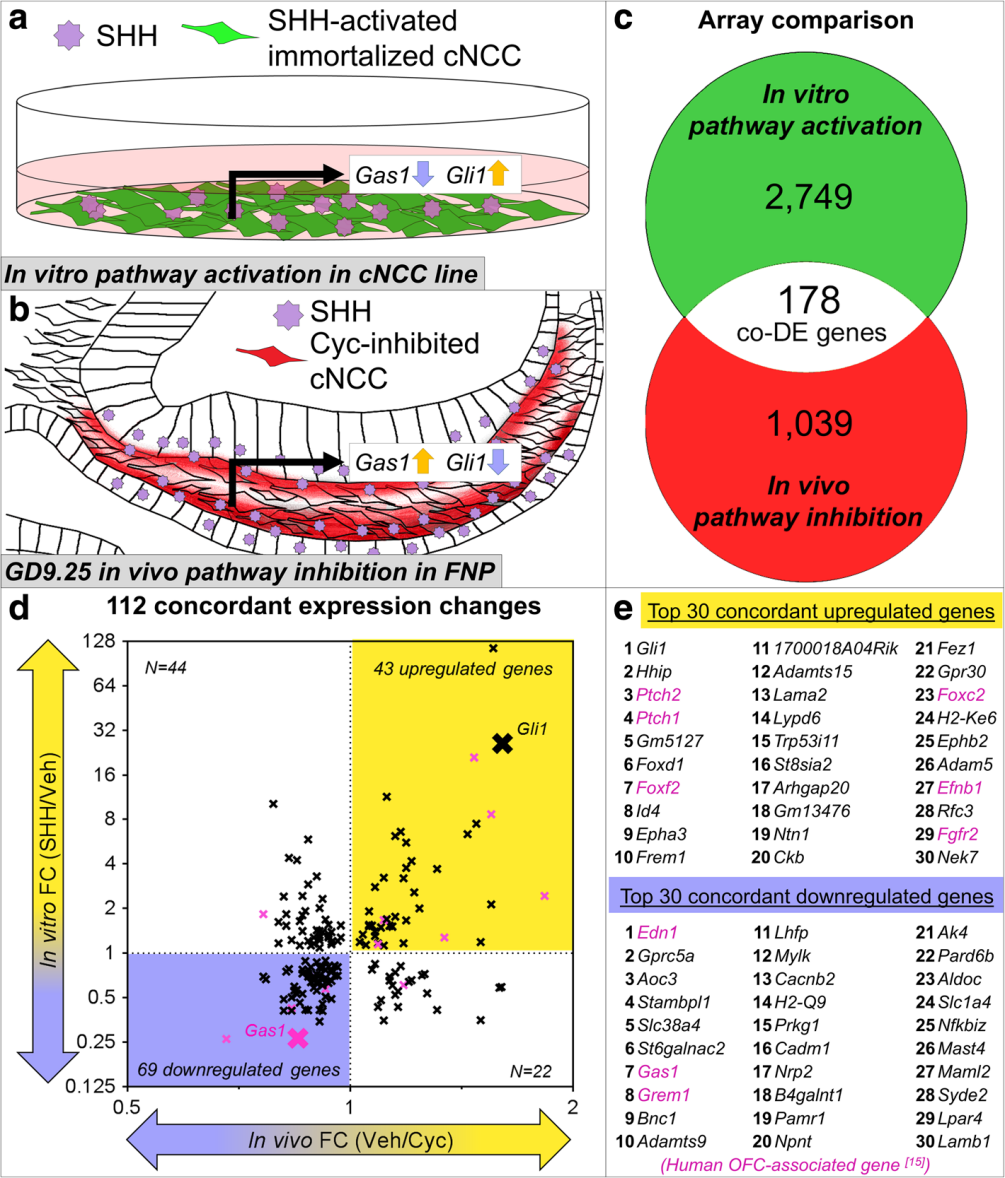
Comparative analysis reveals 112 concordant Shh-regulated genes. **a** Schematic of in vitro Shh pathway activation of cNCCs. **b** Schematic of in vivo Shh pathway inhibition by cyclopamine. Red shading marks cNCCs that would normally respond to SHH ligand stimulation but are blocked by cyclopamine. **c** Comparison of the significant differentially expressed genes between the in vitro model of Shh pathway activation (2749 genes) and the in vivo model of Shh-pathway inhibition (1039 genes). 178 differentially expressed genes are common and overlap between the two microarrays. **d** Scatter plot of 178 common genes. Y-axis shows fold change (SHH/Veh) for the in vitro model of Shh pathway activation, while the X-axis shows fold change (Veh/Cyc) for the in vivo model of Shh pathway inhibition. For clarity of results, in vivo fold change is plotted as Veh/Cyc, such that positively regulated Shh targets appear upregulated in the array and negatively regulated Shh targets appear downregulated. 43 genes show concordant upregulation (positive regulation by Shh signaling, yellow), and 69 genes show concordant downregulation (negatively regulated by Shh signaling, blue). The bona fide Shh targets *Gli1* and *Gas1* are highlighted. Known orofacial cleft (OFC)-associated genes, including *Gas1*, are marked by magenta X’s [15]. **e** The top 30 concordant up- and downregulated genes are listed by rank sum, with OFC-associated genes in magenta. See Table 1 and Table 2 for specific fold changes and FDR *p*-values and Additional files 5 and 6 for gene ranking. A full list of the 112 concordant Shh-regulated genes and the 67 genes with non-concordant changes with their respective linear fold changes can be found in Additional file 4. In vivo array utilized stage-matched FNP tissue from *n* = 6 vehicle and n = 6 cyclopamine-exposed pooled litters

To complement the set of Shh-targets identified in cultured cNCCs (Fig. 4a), we integrated a separate set of 1039 (599 upregulated and 440 downregulated) genes dysregulated in midfacial primordia following Shh pathway inhibition (Fig. 4b and Additional file 3). In this model, transient exposure to the Shh pathway inhibitor cyclopamine from gestational day (GD)8.25–9.25 results in orofacial clefts that mimic human phenotypes [10, 13, 14]. FNP tissue was microdissected at GD9.25, and gene expression changes were determined by comparing to vehicle-exposed control samples [13]. While transient pathway inhibition in this tissue comprised of multiple cell types elicited less robust transcriptional differences, this model captured gene expression changes during the pathogenesis of clinically-relevant OFC outcomes. Comparison of these two data sets allowed for the identification of Shh-regulated genes expressed in the cNCC mesenchyme that are also dysregulated during the initial pathogenesis of orofacial clefting. One hundred seventy-eight common genes were significantly differentially expressed in both arrays (Fig. 4c). These overlapping genes were filtered based on direction of change, with the assumption that a true Shh-regulated gene that is upregulated with the addition of SHH ligand in vitro will be downregulated in vivo with exposure to the Shh pathway inhibitor cyclopamine (or vice versa). Concordant expression changes were observed for 112 genes of the 178 overlapping genes (Fig. 4d and Additional file 4). These positively (*n* = 43) and negatively (*n* = 69) Shh-regulated genes include 14 genes associated with OFCs in humans [15]. The top 30 concordant up- or downregulated genes are listed in Fig. 4e, Tables 1 and 2, and all genes with either concordant or non-concordant expression changes are listed in Additional file 4. Gene ranking is shown in Additional file 5 for positively-regulated genes and Additional file 6 for negatively-regulated genes.

**Table 1:** Top positively Shh-regulated concordant genes

Rank	Gene Symbol	In vitro FC (SHH/Veh)	In vitro FDR *p*-value	In vivo FC (Veh/Cyc)	In vivo FDR *p*-value
1	*Gli1*	25.62	0.000009	1.61	0.104969
2	*Hhip*	114.91	0.000001	1.56	0.119514
3	*Ptch2*	8.68	0.000009	1.55	0.006306
4	*Ptch1*	21	8.20E-07	1.47	0.007005
5	*Gm5127*	7.49	0.000117	1.48	0.538991
6	*Foxd1*	6.37	0.000056	1.44	0.380027
7	*Foxf2*	2.43	0.000041	1.83	0.175736
8	*Id4*	2.13	0.000259	1.55	0.723333
9	*Epha3*	3.7	0.00002	1.31	0.668124
10	*Frem1*	4.18	0.000039	1.21	0.442643
11	*1700018A04Rik*	5.58	0.000086	1.19	0.698151
12	*Adamts15*	6.61	0.000038	1.17	0.704734
13	*Lama2*	3.76	0.00002	1.19	0.571271
14	*Lypd6*	11.4	0.000125	1.12	0.737329
15	*Trp53i11*	2.57	0.000119	1.22	0.516635
16	*St8sia2*	6.19	0.000014	1.15	0.488058
17	*Arhgap20*	2	0.004416	1.24	0.529114
18	*Gm13476*	3.2	0.000017	1.18	0.641684
19	*Ntn1*	3.23	0.000133	1.11	0.618111
20	*Ckb*	1.19	0.000728	1.5	0.447282
21	*Fez1*	1.66	0.00097	1.19	0.704734
22	*Gpr30*	1.76	0.000049	1.15	0.704734
23	*Foxc2*	1.28	0.004471	1.34	0.442166
24	*H2-Ke6*	1.69	0.001836	1.12	0.516635
25	*Ephb2*	2.79	0.000911	1.08	0.73884
26	*Adam5*	1.53	0.009818	1.13	0.704734
27	*Efnb1*	1.67	0.000085	1.11	0.618111
28	*Rfc3*	1.51	0.000725	1.14	0.41917
29	*Fgfr2*	1.67	0.000309	1.11	0.689333
30	*Nek7*	1.17	0.003294	1.18	0.618111

*FC* fold change

**Table 2:** Top negatively Shh-regulated concordant genes

Rank	Gene Symbol	In vitro FC (SHH/Veh)	In vitro FDR *p*-value	In vivo FC (Veh/Cyc)	In vivo FDR *p*-value
1	*Edn1*	0.262467192	0.000042	0.680272109	0.195648
2	*Gprc5a*	0.408163265	0.000156	0.81300813	0.730671
3	*Aoc3*	0.259067358	0.00002	0.847457627	0.725263
4	*Stambpl1*	0.409836066	0.000181	0.833333333	0.654448
5	*Slc38a4*	0.408163265	0.004794	0.833333333	0.737329
6	*St6galnac2*	0.487804878	0.00094	0.81300813	0.714645
7	*Gas1*	0.261096606	0.000085	0.854700855	0.749637
8	*Grem1*	0.423728814	0.000056	0.833333333	0.641684
9	*Bnc1*	0.502512563	0.000752	0.833333333	0.226873
10	*Adamts9*	0.462962963	0.000004	0.854700855	0.448794
11	*Lhfp*	0.558659218	0.000247	0.840336134	0.723333
12	*Mylk*	0.529100529	0.000042	0.847457627	0.32826
13	*Cacnb2*	0.414937759	0.000106	0.869565217	0.618111
14	*H2-Q9*	0.564971751	0.00101	0.847457627	0.170192
15	*Prkg1*	0.595238095	0.00159	0.840336134	0.488058
16	*Cadm1*	0.411522634	0.000029	0.877192982	0.749129
17	*Nrp2*	0.666666667	0.000395	0.769230769	0.685182
18	*B4galnt1*	0.694444444	0.000643	0.763358779	0.560523
19	*Pamr1*	0.531914894	0.004349	0.862068966	0.723333
20	*Npnt*	0.344827586	0.000037	0.909090909	0.448794
21	*Ak4*	0.41322314	0.000639	0.909090909	0.158579
22	*Pard6b*	0.406504065	0.000074	0.917431193	0.707119
23	*Aldoc*	0.751879699	0.001536	0.819672131	0.618111
24	*Slc1a4*	0.584795322	0.002363	0.892857143	0.730671
25	*Nfkbiz*	0.636942675	0.000028	0.884955752	0.661155
26	*Mast4*	0.558659218	0.000588	0.900900901	0.618111
27	*Maml2*	0.454545455	0.000009	0.917431193	0.618111
28	*Syde2*	0.78125	0.007317	0.826446281	0.704734
29	*Lpar4*	0.689655172	0.000181	0.877192982	0.317827
30	*Lamb1*	0.694444444	0.000362	0.877192982	0.155497

*FC* fold change

**Fig. 5:**
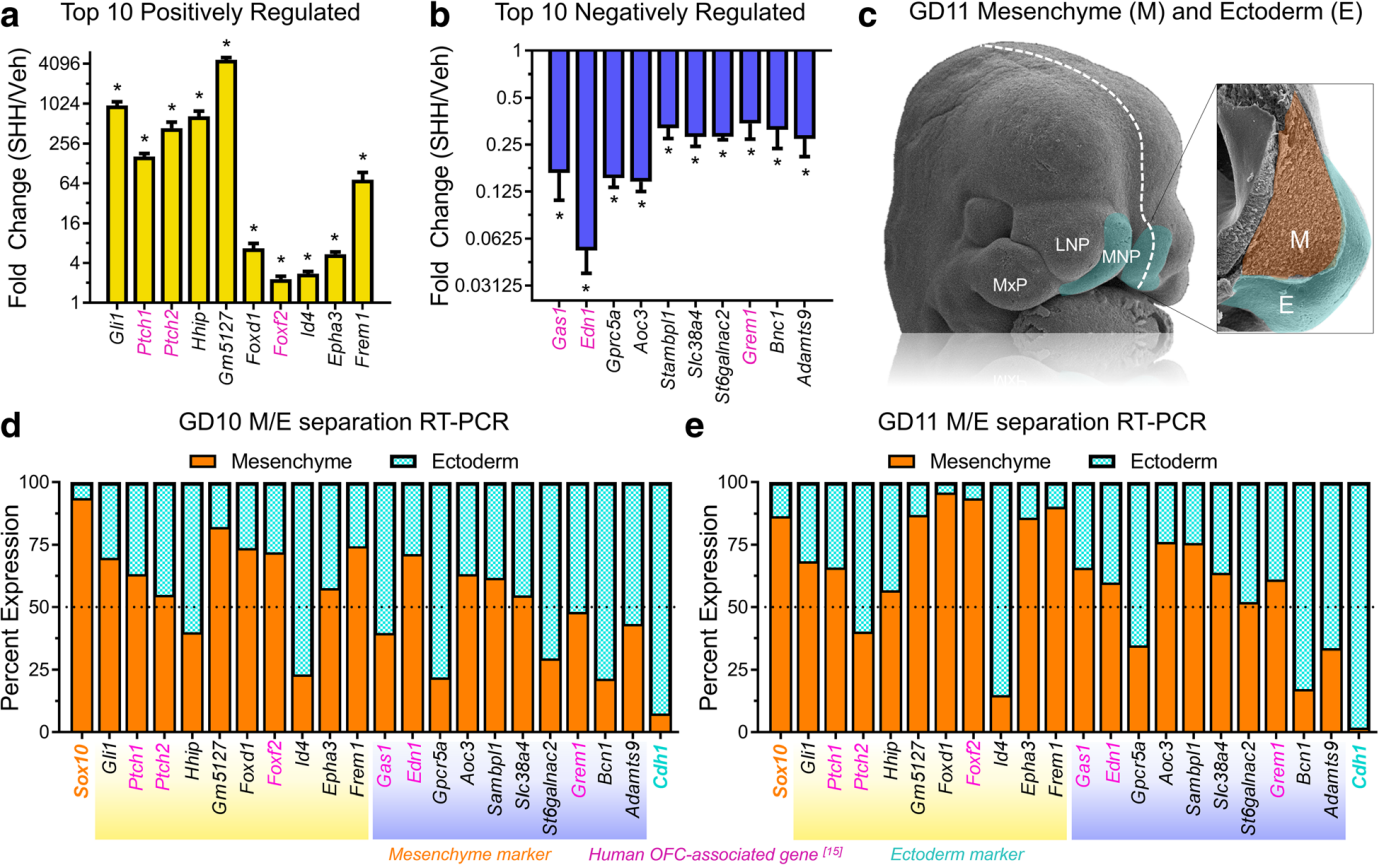
Expression validation of concordant Shh-regulated genes. **a**-**b** Vehicle-normalized expression relative to *Gapdh* is shown for the top 10 (**a**) up- and (**b**) downregulated genes with concordant expression changes. Mean fold change (SHH/Veh) +/− SEM is shown. Known orofacial cleft-associated genes in humans are highlighted in magenta [15]. *N* = 4 samples per treatment group. **c** Pseudo-colored scanning electron micrographs are shown for an intact and a sagittally cut (white dashed line) GD11 mouse embryo. The cranial neural crest-derived mesenchyme (orange) and surface ectoderm (teal) of the MNPs of GD10 or GD11 embryos were separated and isolated for RT-PCR analysis. **d**-**e** Tissue-specific expression enrichment was determined for the top 10 up- and downregulated concordant genes. Values represent the mean expression per *Gapdh* for the mesenchymal (orange) and ectodermal (teal) compartments of the MNPs for each gene divided by the total expression in both tissues multiplied by 100%. *Sox10* (orange) is a specific marker of the cNCC-derived mesenchyme, and *Cdh1* (E-cadherin, teal) is a specific marker of the surface ectoderm. Surface ectoderm and mesenchyme tissues were isolated from *N* = 3 pooled litters per gestational stage. MNP = medial nasal process, LNP = lateral nasal process, MxP = maxillary process, M = mesenchyme, E = ectoderm, OFC = orofacial cleft

We next sought to validate the differential expression of the top 10 genes positively and negatively regulated by Shh signaling. RT-PCR confirmed significant differential expression and direction of change for each of these genes in response to SHH ligand stimulation in vitro (Fig. 5a, b). Similarly, each of the top 10 positively regulated genes was significantly reduced by pathway inhibition in vivo (Additional file 7). Next, to determine the tissue localization for each of the top Shh-regulated genes during normal orofacial development, mesenchyme and surface ectoderm of the tissues that form the median upper lip, the paired medial nasal processes (MNPs), of GD10 or GD11 embryos were enzymatically separated and isolated [16] (Fig. 5c). Gene expression was then analyzed for each tissue compartment by RT-PCR and reported as the percent of total expression in the mesenchyme and ectoderm (see methods). Expression for the majority of the top 20 Shh-regulated genes was enriched in the mesenchyme of the MNPs at GD10 (12/20) and GD11 (15/20) (Fig. 5d, e).

**Fig. 6:**
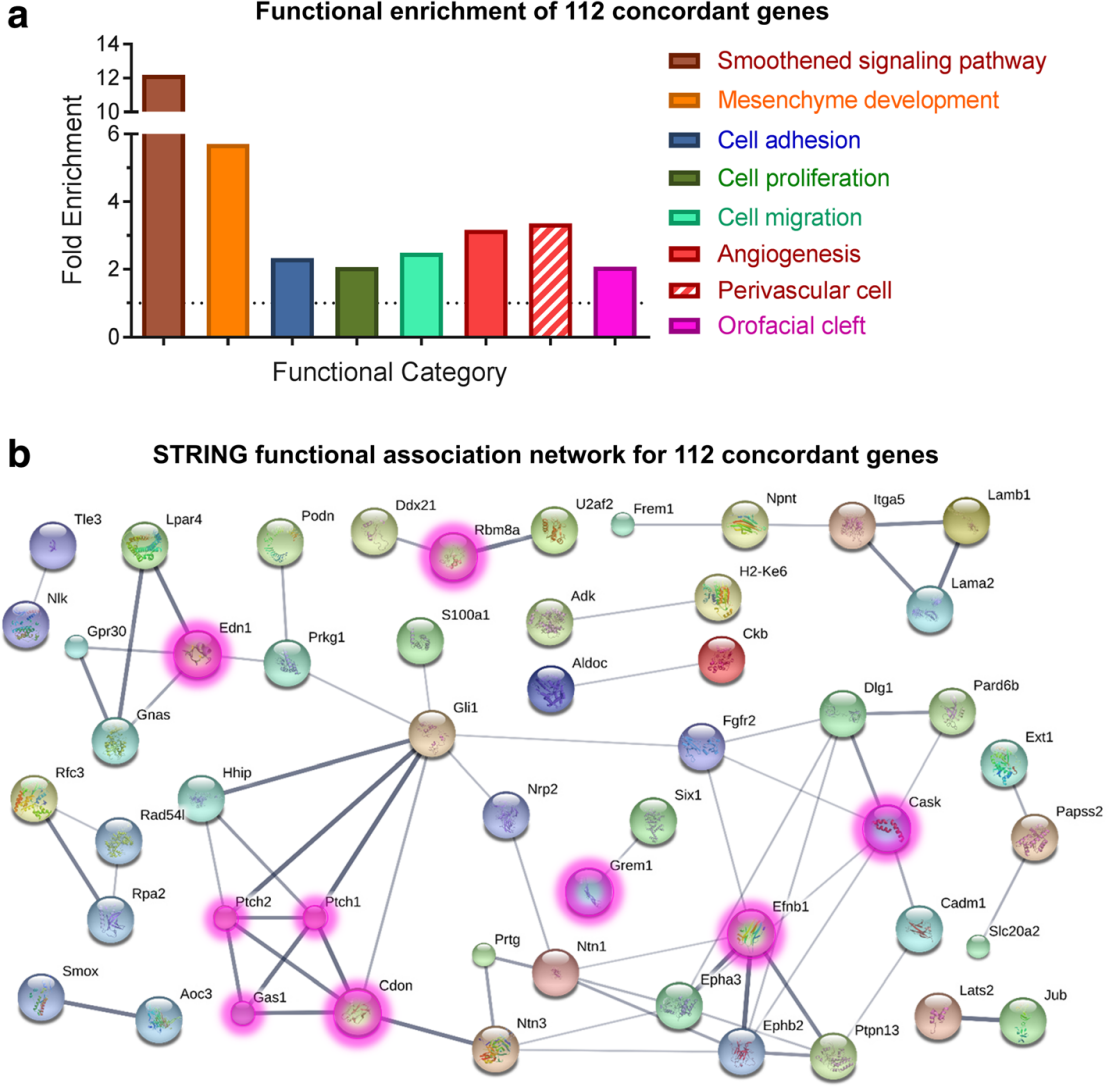
Enrichment analysis of 112 concordant Shh-regulated genes. **a** Functional enrichment analysis of 112 genes with concordant expression changes in the two microarrays. A table of these results is available as Additional file 8. **b** Predicted protein interaction network of 112 genes with concordant expression changes. Known orofacial cleft (OFC)-associated genes in humans are highlighted in magenta [15]

Next, to describe the possible roles of Shh-regulated genes in cNCCs, functional gene annotation enrichment analysis was conducted on the 112 genes that were Shh-regulated in vivo and in vitro (Fig. 6a and Additional file 8). Serving as a proof of concept, Smoothened signaling pathway (fold enrichment = 12.19, *p*-value = 7.32 × 10^− 4^) and mesenchyme development (fold enrichment = 5.709, *p*-value = 4.88 × 10^− 4^) were each significantly enriched in the Shh-regulated gene set. More interestingly, significant enrichment of biological processes critical for cNCC biology and orofacial development was also observed, including cell adhesion (fold enrichment = 2.329, *p*-value = 4.79 × 10^− 4^), cell proliferation (fold enrichment = 2.061, *p*-value = 1.60 × 10^− 3^), cell migration (fold enrichment = 2.489, *p*-value = 1.07 × 10^− 3^), angiogenesis (fold enrichment = 3.163, *p*-value = 1.28 × 10^− 2^), and perivascular cell markers (fold enrichment = 3.355, *p*-value = 7.65 × 10^− 7^) (genes in perivascular cell category are listed in Additional file 8). Based on previous findings that Shh-regulated genes in the cNCC-derived mesenchyme promote the tissue outgrowth required for proper upper lip morphogenesis [13], we predicted a significant proportion of human OFC-associated genes would be regulated by Shh signaling in the mesenchyme of the FNP and/or MNPs. Therefore, a category was constructed based on a recently published compilation of human OFC-associate genes [15], and the overrepresentation of those genes was assessed in the Shh-regulated gene set (genes in OFC category are listed in Additional file 8 and [15]). Interestingly, OFC-associated genes were significantly enriched among Shh-regulated genes, suggesting that many known OFC genes are Shh-regulated in the cNCC-derived mesenchyme (fold enrichment = 2.077; *p*-value = 2.82 × 10^− 7^) (Additional file 8). Lastly, a protein interaction network was produced showing known and proposed interactions between the 112 concordant genes and highlighting the large number of Shh-regulated genes that are already-described human OFC genes (Fig. 6b).

## Discussion

The cranial neural crest is a multipotent cell population that migrates from the dorsal margins of the neural folds to populate the facial primordia and pharyngeal arches by GD9.0 in the mouse [17]. Following migration, these cells receive developmental cues from the surrounding neuroectoderm and surface ectoderm that guide their proliferation and differentiation into the facial bones, cartilage, and the neurons and glia of the peripheral nervous system [18, 19]. While SHH is considered a major regulator of epithelial-mesenchymal interactions in craniofacial morphogenesis, the specific biological processes and intermediary target genes that regulate these processes are not fully understood.

We compared the transcriptional response of isolated cNCCs exposed to SHH ligand to that of primordial midfacial tissue exposed to a specific Shh pathway inhibitor to reveal new insight into the role of Shh signaling in cNCC biology and OFC pathogenesis. While ensuring that the observed expression changes were specific to the cNCC-derived mesenchyme, the in vitro system yielded robust expression changes, maximizing our ability to identify Shh-regulated genes. The in vivo model of transient pathway inhibition in FNP tissue comprised of multiple cell types elicited more modest transcriptional changes but provided a filter to enrich for genes relevant to cleft pathogenesis [10]. Leveraging and integrating the unique advantages of these models enabled the identification of Shh-regulated genes specifically in the cNCC-derived mesenchyme that are also dysregulated during the initial pathogenesis of clinically relevant OFC outcomes.

Our findings suggest Shh signaling has pleiotropic effects on cNCC biology. Several biological processes that cNCCs normally undergo during embryogenesis, which are required for proper orofacial development, appeared to be regulated by Shh signaling in cNCCs, including cell migration, cell proliferation, and cell adhesion [20, 21]. A surprising finding from this study was the identification of angiogenesis as a Shh-regulated process in cNCCs during OFC pathogenesis. While Shh signaling has been linked to angiogenesis by several lines of investigation, the underlying mechanism is controversial [22–24]. For example, some reports argue that SHH acts directly on endothelial cells, while others suggest that endothelial cells are not capable of a canonical response to SHH ligand [22, 25]. Importantly, lineage tracing experiments have demonstrated that multipotent cNCCs give rise to the perivascular cells/pericytes of the head and face [26], but the molecular drivers of their differentiation have not been identified. We show that Shh regulates key angiogenic and perivascular genes in isolated cNCCs, and that Shh regulation of angiogenic and perivascular genes is disrupted during the initial pathogenesis of cleft lip. These results suggest that Shh signaling may promote the differentiation of multipotent cNCCs to endothelium-supporting perivascular cells/pericytes during craniofacial morphogenesis, and disruption of this molecular-cellular program may contribute to OFC pathogenesis. Despite the requirement for new vasculature formation during tissue outgrowth, disruption of angiogenesis is a relatively understudied mechanism in orofacial clefting. The demonstration that Shh signaling regulates angiogenic and perivascular genes in the cNCC-mesenchyme of the developing upper lip provides new opportunities to investigate the complex mechanisms underlying orofacial clefting.

Morphogenesis of the upper lip and secondary palate share molecular drivers and cellular components, including a densely packed cNCC-derived mesenchyme [8, 27]. However, the etiology and pathogenesis of cleft lip is often considered distinct from that of cleft palate [28–32]. While cleft lip is one of the most common structural malformations in humans and the most common OFC subtype [33], our understanding of its pathogenesis has been limited by the relative resistance of mice to this malformation. Here, we leveraged one of the few mouse models of non-syndromic cleft lip to identify cNCC targets of Shh signaling during initial cleft lip pathogenesis. In addition to many genes already linked to OFCs in humans, this study highlights new candidates to examine in OFC etiology. In fact, several of the Shh target genes identified here in the cNCC, including *Adamts9, Edn1*, *Dlg1,* and *Efnb1,* are already implicated in cleft palate pathogenesis by existing mouse models*.* Our findings argue that these genes should be specifically examined as candidate human OFC genes in patient populations with either cleft palate or cleft lip.

The majority of OFC cases in humans are of unknown etiology, suggesting additional genes remain to be identified. Using our in vivo system, we previously found the Forkhead box transcription factor *Foxf2* to be a canonical Shh target gene that promotes proliferation of the cNCC-derived mesenchyme and is downregulated in the cNCC-derived FNP and MNP mesenchyme during OFC pathogenesis [13]. Human relevance of this model was reinforced with the identification of *FOXF2* SNPs associated with OFCs in a large, multi-ethnic patient population [13]. Building upon this foundation, the current study demonstrated that 14 genes previously linked to human OFCs are Shh-regulated in cNCCs. This finding bolsters the possibility that several of the novel Shh-regulated genes identified here may contribute to OFC etiology, warranting their examination in human patient populations.

## Conclusions

Orofacial clefts are common congenital malformations that cause significant morbidity in affected children. While we have made great advances in understanding the etiology of these complex human birth defects, the causes of the malformations remain unexplained for the majority of patients. Here, we utilized a novel approach to Shh target gene identification to discover genes in the cNCC-derived mesenchyme that are Shh-regulated and whose expression is altered during the initial pathogenesis of OFCs. We identified several known and novel biological processes that appear Shh-regulated in cNCCs and dysregulated during OFC pathogenesis. Together, this work improves our understanding of the roles of Shh signaling in cNCC biology and identifies several novel Shh-regulated genes as candidate OFC genes, providing new avenues to elucidate the basis of these common human birth defects.

## Methods

### In vitro cell culture

Immortalized O9–1 cranial neural crest cells were provided by Dr. Robert Maxson, Keck School of Medicine at the University of Southern California and cultured as described by Ishii and colleagues [12] with the following modifications: 0.25% trypsin in 0.5 mM EDTA was used for passaging at a subculture ratio of 1:3 to 1:10. Cells were allowed to attach for 24 h and media were replaced with DMEM containing 1% FBS ± SHH-N peptide (R&D Systems, Minneapolis, MN) at 0.4 μg/mL. 48 h after SHH treatment, cells were lysed for RNA collection. *N* = 4 biological replicates were examined in each treatment group for microarray and RT-PCR analyses.

### RNA extraction and purification

RNA was isolated using GE Illustra RNAspin kits (GE Healthcare). RNA quality was determined by analysis with an Agilent Bioanalyzer 2100 (Additional file 9). This RNA starting material was used for both microarray analysis and RT-PCR validation assays. cDNA was synthesized from 250 μg of total RNA using Promega GoScript (Promega Corporation) reverse transcription reaction kits.

### Microarray sample preparation, hybridization, and scanning

Affymetrix Mouse Gene 2.0 ST GeneChip® arrays were used for microarray analysis. Ambion Whole-Transcript Expression and the Affymetrix GeneChip Whole Transcript Terminal Labeling Reagent kits were employed to generate sense strand end-terminus biotin-labeled single-stranded cDNA. 100-500 ng of total RNA was used for input in a first strand cDNA synthesis reaction to specifically prime non-ribosomal RNA, including both poly(A) and non-poly(A) mRNA, followed immediately by second strand cDNA synthesis. The 2nd cDNA was purified with paramagnetic beads and input into an 8 h IVT reaction to generate amplified RNA. This amplified RNA was then input into the 2nd cycle cDNA synthesis to generate double-stranded cDNA, which was purified by paramagnetic beads, fragmented, and end-terminus labeled with biotin. The end-terminus, biotin-labeled cDNA was hybridized to an AFX Mouse Gene 2.0 ST GeneChip for 16 h at 45 °C (one sample per chip for a total of 8 chips). Following hybridization, the GeneChip was washed and stained with Streptavidin-Phycoerythrin (SAPE) on the AFX Fluidics450 Station. To quantify the fluorescent signal from each feature on the GeneChip, all GeneChips were scanned at a wavelength of 570 nm on the GC3000 G7 scanner. Fluorescent signals corresponding to hybridization intensities were determined using the Affymetrix GCOS software. Intensity files were then converted to signal files using Affymetrix Expression Console for subsequent analysis.

### Microarray computational analysis

Significant differential gene expression was determined using Affymetrix Transcriptome Analysis Console (TAC, v.2.0.0.9). Gene annotation enrichment analysis for pathways and biological processes was conducted using the Database for Annotation, Visualization and Integrated Discovery (DAVID) [34, 35]. The orofacial cleft gene set was compiled based on Funoto et al. [15], and the perivascular cell gene set was compiled based on He et al. [36]. Gene lists for both categories are presented in Additional file 8. Identification of top differentially expressed genes in the comparative analysis was accomplished by ranking each concordant gene by fold change in both the in vivo and in vitro arrays and calculating the rank sum for each gene. The top 10 positively and negatively regulated genes were selected for additional analysis.

### Mesenchyme-ectoderm separation

Separation of mesenchyme and surface ectoderm of GD10 and GD11 medial nasal processes was accomplished as previously described [13, 16]. Expression per *Gapdh* was calculated for each gene in each tissue, and the percent expression in the epithelial and mesenchymal compartment vs total expression (sum expression in both compartments) is reported.

### Quantitative real-time RT-PCR

Singleplex RT-PCR was conducted as previously described [37] using SSoFast EvaGreen Supermix (BioRad Laboratories) and a BioRad CFX96 Touch thermocycler. Gene-specific RT-PCR primers were designed using IDT PrimerQuest (http://www.idtdna.com/primerquest). Primers were resuspended as stock solutions of 100 μM in TE buffer at pH = 7.0 (Ambion). Working stocks were made as 10 μM solutions containing both forward and reverse primers. Primer sequences are listed in Additional file 10. *Gapdh* was used as the housekeeping gene and analyses were conducted with the 2^-ddCt method.

### Statistics

Affymetrix Transcriptome Analysis Console (TAC) v. 2.0.0.9 was used for determination of significant differential expression in microarray experiments. Due to inherent differences between the samples used for each analysis (i.e. a uniform cell population with Shh pathway activation vs complex in vivo tissue with transient Shh pathway perturbation), an FDR *p*-value (Benjamini-Hochberg *p*-value) less than 0.01 was utilized for the in vitro microarray and an FDR *p*-value less than 0.75 was used for the in vivo microarray for determination of significance. This strategy was chosen to minimize false negatives for the less robust in vivo array, while false positives are reduced by comparing to the more stringently gated in vitro data set. The Database for Annotation, Visualization and Integrated Discovery (DAVID) was used for GO and INTERPRO enrichment analyses of differentially expressed genes [34, 35]. Enrichment for orofacial cleft and perivascular cell markers were assessed by a hypergeometric distribution test using categories built from published gene sets [15, 36] (genes for each category are listed in Additional file 8), with an alpha value < 0.05 considered significant. Two-tailed t-tests with Holm-Sidak correction were used for RT-PCR experiments using *Graphpad Prism 6*, with an alpha value < 0.05 considered significant.

## Additional files

Additional file 1: Table of all genes with respective expression data for in vitro microarray analysis. (XLS 36513 kb)

Additional file 2: Table of enriched biological categories for in vitro analysis. (XLS 36 kb)

Additional file 3: Table of all genes with respective expression data for in vivo microarray analysis. (XLS 6587 kb)

Additional file 4: Table of common genes dysregulated in both in vivo and in vitro microarrays with respective expression data. Concordant genes are marked in the last column. (XLS 93 kb)

Additional file 5: Ranking of positively regulated concordant genes for Fig. 4e. (XLS 44 kb)

Additional file 6: Ranking of negatively regulated concordant genes for Fig. 4e. (XLS 46 kb)

Additional file 7: RT-PCR validation of positively Shh-regulated genes in vivo. (PDF 113 kb)

Additional file 8: Table of enriched biological categories for concordant genes. (XLS 80 kb)

Additional file 9: Agilent Bioanalyzer 2100 RNA quality results for in vitro and in vivo samples. (PDF 215 kb)

Additional file 10: Table of RT-PCR primer sequences. (XLS 37 kb)

## Abbreviations

Cyc: Cyclopamine
DE: Differentially expressed
E: Ectoderm
FC: Fold change
FNP: Frontonasal prominence
GD: Gestational day
LNP: Lateral nasal process
M: Mesenchyme
MNP: Medial nasal process
MxP: Maxillary process
OFCs: Orofacial clefts
SHH: Shh ligand/protein
Shh: Sonic Hedgehog
Veh: Vehicle

## References

[1] Tolarová MM, Cervenka J (1998). Classification and birth prevalence of orofacial clefts. Am J Med Genet.

[2] Murray JC (2002). Gene/environment causes of cleft lip and/or palate. Clin Genet.

[3] Edison R, Muenke M (2003). The interplay of genetic and environmental factors in craniofacial morphogenesis: holoprosencephaly and the role of cholesterol. Congenit Anom (Kyoto).

[4] Juriloff DM, Harris MJ (2008). Mouse genetic models of cleft lip with or without cleft palate. Birth Defects Res A Clin Mol Teratol.

[5] Vieira AR (2008). Unraveling human cleft lip and palate research. J Dent Res.

[6] Graham JM, Shaw GM (2005). Gene-environment interactions in rare diseases that include common birth defects. Birth Defects Res A Clin Mol Teratol.

[7] Oski FA (1990). Principles and practice of pediatrics.

[8] Jiang R, Bush JO, Lidral AC (2006). Development of the upper lip: morphogenetic and molecular mechanisms. Dev Dyn.

[9] Marcucio RS, Cordero DR, Hu D, Helms JA (2005). Molecular interactions coordinating the development of the forebrain and face. Dev Biol.

[10] Lipinski RJ, Song C, Sulik KK, Everson JL, Gipp JJ, Yan D, Bushman W, Rowland IJ (2010). Cleft lip and palate results from hedgehog signaling antagonism in the mouse: phenotypic characterization and clinical implications. Birth Defects Res A Clin Mol Teratol.

[11] Cordero D, Marcucio R, Hu D, Gaffield W, Tapadia M, Helms JA (2004). Temporal perturbations in sonic hedgehog signaling elicit the spectrum of holoprosencephaly phenotypes. J Clin Invest.

[12] Ishii M, Arias AC, Liu L, Chen YB, Bronner ME, Maxson RE (2012). A stable cranial neural crest cell line from mouse. Stem Cells Dev.

[13] Everson JL, Fink DM, Yoon JW, Leslie EJ, Kietzman HW, Ansen-Wilson LJ, Chung HM, Walterhouse DO, Marazita ML, Lipinski RJ (2017). Sonic hedgehog regulation of Foxf2 promotes cranial neural crest mesenchyme proliferation and is disrupted in cleft lip morphogenesis. Development.

[14] Lipinski RJ, Hutson PR, Hannam PW, Nydza RJ, Washington IM, Moore RW, Girdaukas GG, Peterson RE, Bushman W (2008). Dose-and route-dependent teratogenicity, toxicity, and pharmacokinetic profiles of the hedgehog signaling antagonist cyclopamine in the mouse. Toxicol Sci.

[15] Funato N, Nakamura M (2017). Identification of shared and unique gene families associated with oral clefts. Int J Oral Sci.

[16] Li H, Williams T. Separation of mouse embryonic facial ectoderm and mesenchyme. J Vis Exp. 2013;74:50248. 10.3791/50248 PMC3654862 23603693

[17] Minoux M, Rijli FM (2010). Molecular mechanisms of cranial neural crest cell migration and patterning in craniofacial development. Development.

[18] Le Douarin NM, Brito JM, Creuzet S (2007). Role of the neural crest in face and brain development. Brain Res Rev.

[19] Dupin E, Creuzet S, Le Douarin NM (2006). The contribution of the neural crest to the vertebrate body. Adv Exp Med Biol.

[20] Noden DM, Trainor PA (2005). Relations and interactions between cranial mesoderm and neural crest populations. J Anat.

[21] Cox TC (2004). Taking it to the max: the genetic and developmental mechanisms coordinating midfacial morphogenesis and dysmorphology. Clin Genet.

[22] Alvarez JI, Dodelet-Devillers A, Kebir H, Ifergan I, Fabre PJ, Terouz S, Sabbagh M, Wosik K, Bourbonnière L, Bernard M, van Horssen J, de Vries HE, Charron F, Prat A (2011). The hedgehog pathway promotes blood-brain barrier integrity and CNS immune quiescence. Science.

[23] Kolesová H, Roelink H, Grim M (2008). Sonic hedgehog is required for the assembly and remodeling of branchial arch blood vessels. Dev Dyn.

[24] Pola R, Ling LE, Silver M, Corbley MJ, Kearney M, Blake Pepinsky R, Shapiro R, Taylor FR, Baker DP, Asahara T, Isner JM (2001). The morphogen sonic hedgehog is an indirect angiogenic agent upregulating two families of angiogenic growth factors. Nat Med.

[25] Renault MA, Roncalli J, Tongers J, Thorne T, Klyachko E, Misener S, Volpert OV, Mehta S, Burg A, Luedemann C, Qin G, Kishore R, Losordo DW (2010). Sonic hedgehog induces angiogenesis via rho kinase-dependent signaling in endothelial cells. J Mol Cell Cardiol.

[26] Etchevers HC, Vincent C, Le Douarin NM, Couly GF (2001). The cephalic neural crest provides pericytes and smooth muscle cells to all blood vessels of the face and forebrain. Development.

[27] Lan Y, Xu J, Jiang R (2015). Cellular and molecular mechanisms of Palatogenesis. Curr Top Dev Biol.

[28] Rahimov F, Jugessur A, Murray JC (2012). Genetics of nonsyndromic orofacial clefts. Cleft Palate Craniofac J.

[29] FRASER FC (1955). Thoughts on the etiology of clefts of the palate and lip. Acta Genet Stat Med.

[30] Sivertsen A, Wilcox AJ, Skjaerven R, Vindenes HA, Abyholm F, Harville E, Lie RT (2008). Familial risk of oral clefts by morphological type and severity: population based cohort study of first degree relatives. BMJ.

[31] Ludwig KU, Mangold E, Herms S, Nowak S, Reutter H, Paul A, Becker J, Herberz R, AlChawa T, Nasser E, Böhmer AC, Mattheisen M, Alblas MA, Barth S, Kluck N, Lauster C, Braumann B, Reich RH, Hemprich A, Pötzsch S, Blaumeiser B, Daratsianos N, Kreusch T, Murray JC, Marazita ML, Ruczinski I, Scott AF, Beaty TH, Kramer FJ, Wienker TF, Steegers-Theunissen RP, Rubini M, Mossey PA, Hoffmann P, Lange C, Cichon S, Propping P, Knapp M, Nöthen MM (2012). Genome-wide meta-analyses of nonsyndromic cleft lip with or without cleft palate identify six new risk loci. Nat Genet.

[32] Leslie EJ, Marazita ML (2013). Genetics of cleft lip and cleft palate. Am J Med Genet C Semin Med Genet.

[33] Watkins SE, Meyer RE, Strauss RP, Aylsworth AS (2014). Classification, epidemiology, and genetics of orofacial clefts. Clin Plast Surg.

[34] Huang dW, Sherman BT, Lempicki RA (2009). Systematic and integrative analysis of large gene lists using DAVID bioinformatics resources. Nat Protoc.

[35] Huang dW, Sherman BT, Lempicki RA (2009). Bioinformatics enrichment tools: paths toward the comprehensive functional analysis of large gene lists. Nucleic Acids Res.

[36] He L, Vanlandewijck M, Raschperger E, Andaloussi Mäe M, Jung B, Lebouvier T, Ando K, Hofmann J, Keller A, Betsholtz C (2016). Analysis of the brain mural cell transcriptome. Sci Rep.

[37] Heyne GW, Everson JL, Ansen-Wilson LJ, Melberg CG, Fink DM, Parins KF, Doroodchi P, Ulschmid CM, Lipinski RJ (2016). Gli2 gene-environment interactions contribute to the etiological complexity of holoprosencephaly: evidence from a mouse model. Dis Model Mech.

