# Supplementary material for: Identification of sonic hedgehog-regulated genes and biological processes in the cranial neural crest mesenchyme by comparative transcriptomics
Source: BMC Genomics. 2018 Jun 27;19:497. doi: 10.1186/s12864-018-4885-5 (PMC6020285; doi:10.1186/s12864-018-4885-5)
Supplement: Supplementary file 7 — RT-PCR validation of positively Shh-regulated genes in vivo. (PDF 113 kb) [file 12864_2018_4885_MOESM7_ESM.pdf]

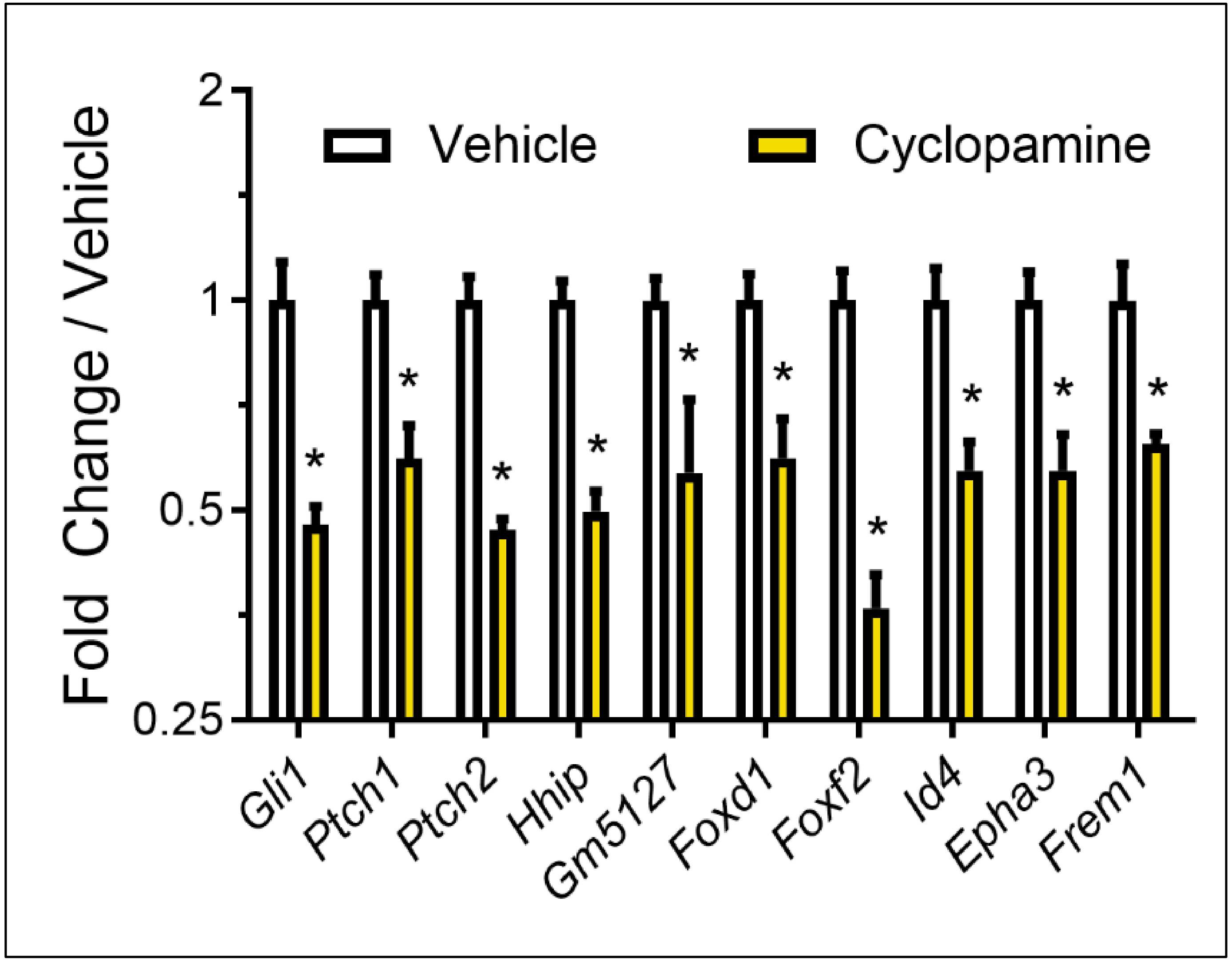

***In vivo* RT-PCR expression validation.** *In vivo* vehicle-normalized expression relative *Gapdh* +/- SEM is shown for the top ten upregulated genes with concordant expression changes. N=6 samples per treatment group. * denotes significant downregulation based on *p*-value < 0.05 by Student's t-test
