# Supplementary material for: Identification of sonic hedgehog-regulated genes and biological processes in the cranial neural crest mesenchyme by comparative transcriptomics
Source: BMC Genomics. 2018 Jun 27;19:497. doi: 10.1186/s12864-018-4885-5 (PMC6020285; doi:10.1186/s12864-018-4885-5)
Supplement: Supplementary file 9 — Agilent Bioanalyzer 2100 RNA quality results for in vitro and in vivo samples. (PDF 215 kb) [file 12864_2018_4885_MOESM9_ESM.pdf]

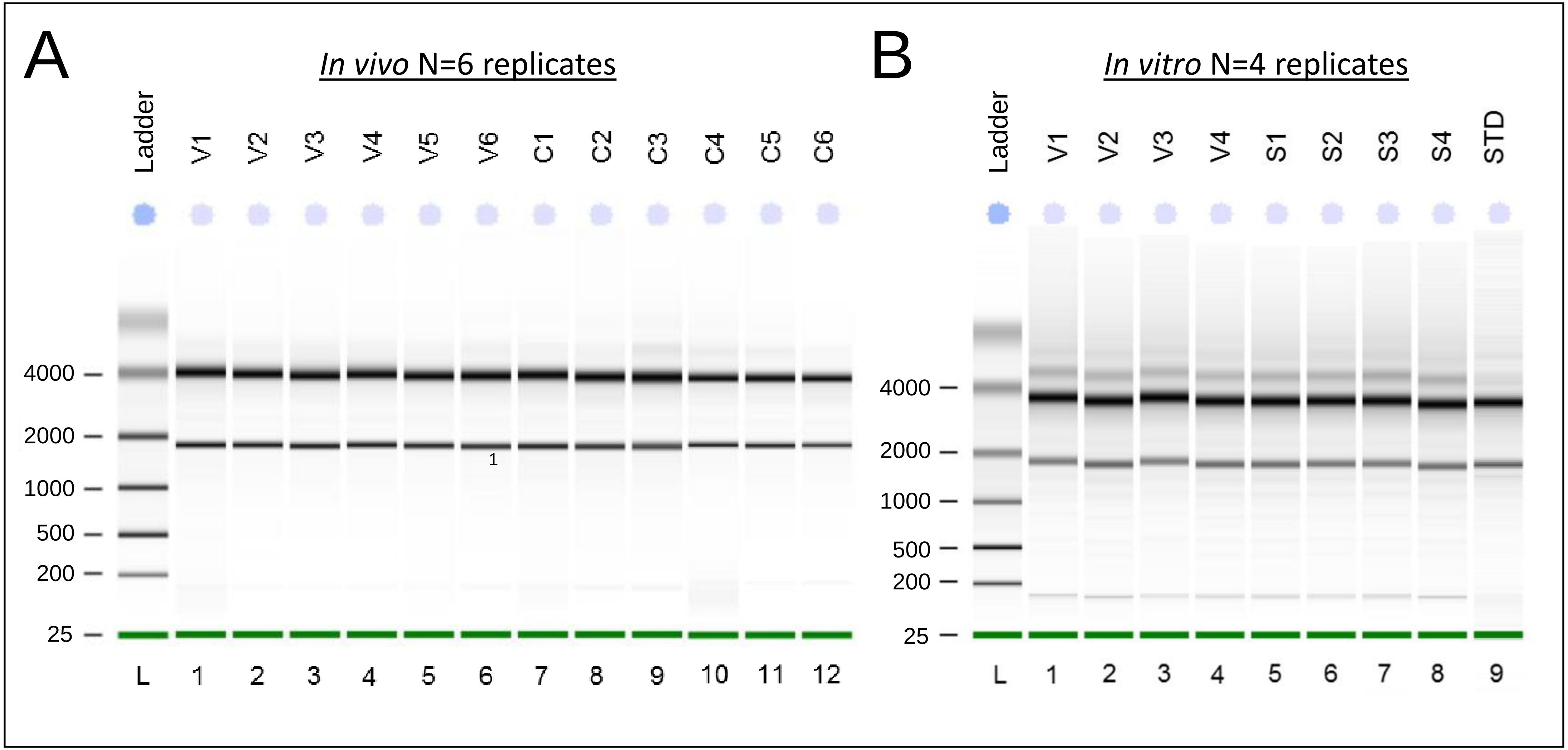

RNA quality assessment. RNA quality was analyzed with an Agilent Bioanlyzer 2100 showing high quality RNA for all (A) 12 *in vivo* samples (V1-V6, C1-C6), (B) 8 *in vitro* samples (V1-V4, S1-S4) and an RNA standard (STD). V=vehicle, C=cyclopamine, S=SHH, L=ladder.
